# Quality assessment of clinical practice in neuro-oncology

**DOI:** 10.31744/einstein_journal/2025AO1343

**Published:** 2025-04-25

**Authors:** Renata Carolina Acri Nunes Miranda, Suzana Maria Fleury Malheiros, André Felix Gentil, Gisele Sampaio Silva, Fabiana de Campos Cordeiro Hirata, Sérgio Eduardo Alonso Araujo, Luís Otávio Sales Caboclo

**Affiliations:** 1 Hospital Israelita Albert Einstein São Paulo SP Brazil Hospital Israelita Albert Einstein, São Paulo, SP, Brazil.; 2 Faculdade Israelita de Ciências da Saúde Albert Einstein Hospital Israelita Albert Einstein São Paulo SP Brazil Faculdade Israelita de Ciências da Saúde Albert Einstein, Hospital Israelita Albert Einstein, São Paulo, SP, Brazil.; 3 Universidade Federal de São Paulo São Paulo SP Brazil Universidade Federal de São Paulo, São Paulo, SP, Brazil.

**Keywords:** Central nervous system neoplasms, Brain neoplasms, Glioma, Meningioma, Patient outcome assessment, Quality improvement, Quality indicators, health care, Length of stay, Postoperative complications, Magnetic resonance imaging

## Abstract

**Objective:**

To evaluate the quality indicators proposed by the American Academy of Neurology and Neuro-oncology Society in patients with primary intracranial central nervous system tumors.

**Methods:**

This study is a retrospective level I analysis that used electronic medical records from the CERNER system and GDOC-SAME at the tertiary hospital. It was approved by the Research Ethics Committee and followed the Declaration of Helsinki. Data was collected and analyzed confidentially via REDCap. The research focused on patients 18 or older with primary central nervous system tumors who had surgery from August 2015 to August 2021. It excluded surgeries performed elsewhere, reoperations, secondary (metastatic) tumors, and primary central nervous system tumors outside the cranium.

**Results:**

The results showed that 48% of the patients had gliomas, whereas 30 and 21.6% had meningiomas and other types of tumors. Quality measures showed that 35% of the patients with grade 2-4 tumors had multidisciplinary care plan discussions. All patients with gliomas underwent molecular testing and those eligible who underwent chemotherapy were educated and provided informed consent. Postoperative magnetic resonance imaging within 72h was performed in 87% of gliomas. The length of hospital stay, postoperative complications, Eastern Cooperative Oncology Group status at discharge, and 30-day status were also potential quality measures. However, for meningioma cases, readmissions were associated with patients in the American Society of Anesthesiologists II (58.33%) and American Society of Anesthesiologists III-IV (41.67%).

**Conclusion:**

The study conclusions revealed that adherence to quality indicators was good; however, improvements are needed in multidisciplinary care plans and postoperative imaging. Quality measures can be enhanced by controlling factors such as American Society of Anesthesiologists and Eastern Cooperative Oncology Group scales at admission, epileptic seizure occurrence, neurological deficits, and tumor size in meningiomas. The study’s findings highlighted the importance of quality improvement programs for optimal medical care.

## In Brief

In this study, we aimed to improve patient care for central nervous system conditions based on the 2021 National Institute for Health and Care Excellence guidelines. Performance measures were used to enhance team accountability and address challenges due to limited evaluate to health insurance and variations in clinical staff decision-making. Rehabilitation and observed variability in surgical costs were not measured in this study. Similar studies were also referenced, and improvements in real-time glioma management were suggested. Performance measures and standardizing practices were recommended to improve cost predictability despite the observed cost variability caused by individualized care.

## Highlights

■ The molecular test according to the WHO classification of CNS tumors (gliomas) was performed in 100% (172) of patients.

■ Education on chemotherapy and informed consent for patients with brain tumors was carried out for 100% (130) of the patients who had an indication for chemotherapy treatment.

■ Approximately 87% of the patients with gliomas underwent postoperative cranial MRI.

■ A multidisciplinary approach, including molecular testing, was used for 35% of patients with glioma.

■ Thromboembolic events after resection of the primary brain tumor occurred in only 0.84% (3) patients.

## Graphical abstract

**Figure:**
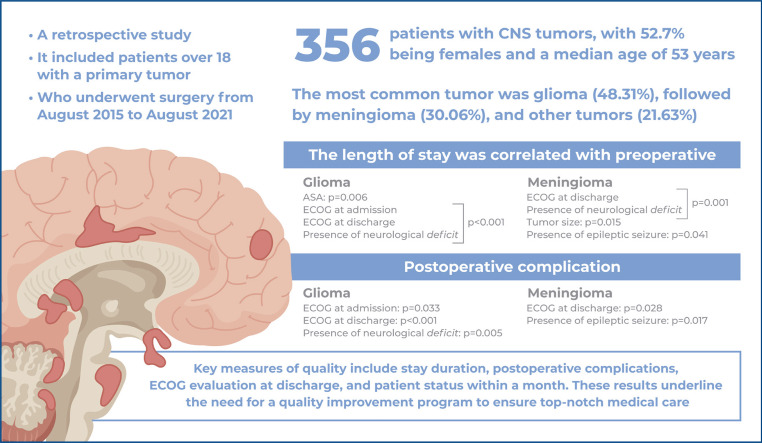

## INTRODUCTION

Primary central nervous system (CNS) tumors, either malignant or not, exhibit symptoms based on their location and growth rate. Meningiomas and gliomas are common in adults, and they account for 80% of malignant CNS tumors and cause high morbidity and mortality rates. These tumors have economic, social, and health effects, including long-term complications and premature death.^1-4^

Multidisciplinary care, which involves various specialties, is essential for patient support during hospitalization, treatment, and follow-up. In addition, comprehensive care improves medical management, planning, diagnosis, and personalized treatment.^4-6^

A unified system for clinical measurements is vital to assessing healthcare quality and improving care and is also essential for health management. Quality indicators are crucial for healthcare providers. These indicators offer resources to identify and disseminate effective practices for treating CNS diseases.^7-12^

The American Society of Clinical Oncology (ASCO)’s practice-based quality assessment program, known for its high-quality cancer education and policy research, emphasizes the importance of quality improvement.^13^

Quality assessments in neuro-oncology are limited; however, incorporating quality indicators can improve practice and outcomes. The American Academy of Neurology (AAN), Society of Neuro-Oncology (SNO), and National Institute for Health and Care Excellence (NICE) suggest using quality measures to enhance practice and outcomes in diagnosing and caring for adult patients with primary and metastatic brain tumors.^14-18^

## OBJECTIVE

To evaluate the quality of neuro-oncology care in a Brazilian tertiary healthcare facility. We focused on patients with primary central nervous system tumors located in the intracranial region who underwent surgery at a high-complexity hospital using the American Academy of Neurology and Society of Neuro-Oncology quality indicators and explored other clinical and surgical variables for quality assessment.

## METHODS

In this level I retrospective study, we used data from the electronic medical records at *Hospital Israelita Albert Einstein* (HIAE). Patients aged >18 years with a primary CNS tumor who underwent surgery at HIAE between August 2015 and August 2021 were included. However, those who underwent surgery elsewhere, had reoperations, metastatic tumors, or extracranial primary CNS tumors were excluded.

Demographic and epidemiological variables included age, sex, and other medical conditions, such as systemic arterial hypertension (SAH), *diabetes mellitus* (DM), dyslipidemia (DLP), smoking, heart disease, pulmonary disease, venous thromboembolism (VTE), and stroke.

Molecular markers were evaluated in patients diagnosed with glioma following the AAN/SNO recommendations.^16^ The histopathological diagnosis was based on the 2016 World Health Organization (WHO) classification,^19^considering that the patient data were included before the WHO’s 2021 updated version.^20^

The tumors were categorized into groups for statistical analysis: gliomas, meningiomas, and other tumors for those that occurred less frequently (<50 times) in our series, such as pituitary adenoma, lymphoma, hemangioblastoma, craniopharyngioma, schwannoma, glioneuronal, medulloblastoma, central mature teratoma, ganglioglioma, hemangiopericytoma, and central neurocytoma.

The collected data that were analyzed based on the primary objective included the quality measures proposed by the AAN/SNO in 2017 (Table 1).

**Table 1:** Set of quality measures developed by the American Academy of Neurology and Neuro-Oncology Society

Metrics	Numerator	Denominator	Exclusion
Multidisciplinary care plan developed for primary tumor	Patients with a multidisciplinary treatment plan developed over a 12-month period	All patients diagnosed in the period with a new diagnosis of CNS tumor - WHO* - grade 2- 4	None
Molecular test according to the WHO classification of SN central tumors (grade 2-4 gliomas)*	Patients with molecular testing performed according to the latest WHO classification flow	Patients aged 18 or over who have had a initial resection or biopsy of glioma grade 2-4	Patients with insufficient tissue for molecular testing
Chemotherapy Education and Informed Consent for Patients with Brain Tumor	Patients who have received chemotherapy education and informed consent obtained prior to prescribing chemotherapy	Patients aged 18 years or older diagnosed with a brain tumor prescribed chemotherapy outside of a clinical trial	Education and consent obtained for the same prescription of chemotherapy in the period of the last 12 months
MRI for intra- and/or postoperative gliomas	Patients who underwent contrast-enhanced MRI intraoperatively or postoperatively (<72h after) surgical resection)	Patients aged 18 and over diagnosed with grade 3-4 glioma who underwent a surgical resection	Patient with surgical clip intracranial or body, neurostimulator, allergy to gadolinium. Patients undergoing surgery for another purposes, except cytoreduction (diagnoses only with biopsy)
VTE Events After Primary Brain Tumor Surgery	Patients who had DVT or pulmonary embolism during your post-surgical hospitalization	All patients who had resection or biopsy of grade 3-4 glioma	DVT or DVT on admission

Source: Translated and adapted from American Academy of Neurology Institute (AANI). Society for Neuro-Oncology (SNO). Neuro-Oncology Quality Measurement Set. U.S.: AANI; SNO; 2023 [cited 2023 Apr 27]. Available from: https://www.aan.com/siteassets/home-page/policy-and-guidelines/quality/quality-measures/18neurooncmeasurementset_pg.pdf^(16)^; * Adapted according to the new WHO 2021 classification: Louis DN, Perry A, Wesseling P, Brat DJ, Cree IA, Figarella-Branger D, et al. The 2021 WHO Classification of Tumors of the Central Nervous System: a summary. Neuro Oncol. 2021;23(8):1231-51. Review.^(20)^

MRI: magnetic resonance imaging; CNS: central nervous system; DVT: deep vein thrombosis; WHO: World Health Organization.

Tumors were classified as intra-axial (gliomas), extra-axial (meningiomas), or other by the Neuro-oncology Medical Assistance Group (GMA) at HIAE based on surgical complexity. Intra-axial tumors were further categorized into superficial, deep, and eloquent areas or the brainstem. Extra-axial tumors were subcategorized based on cranial base location and venous sinus involvement. Tumor size was classified using the GMA score from the preoperative MRI report, categorized as ≥3 or <3cm, or ignored if the MRI was performed elsewhere without a detailed description of the tumor diameter.

Regarding the functional performance, prognosis estimation, and treatment decisions of patients during hospitalization and at medical discharge, the Eastern Cooperative Oncology Group (ECOG) performance status scale ranging from 0-5 was used. The following variables were also analyzed: antiseizure medication use, corticosteroids, and epileptic seizures. Anesthetic risk was assessed using the American Society of Anesthesiologists (ASA) scale, which was used in the pre-anesthetic assessment, with scores ranging from 1-6.^21^

Factors evaluated in the outcome assessment included the length of hospital stay and postoperative complications such as infection, intracranial hemorrhage, neurological deficit, epileptic seizure, hydrocephalus, VTE, diabetes insipidus, hemorrhage, and other less commonly encountered complications. The ECOG score at discharge and the patient’s 30-day status (alive, dead, readmitted, or “not applicable” for in-hospital deaths) were also evaluated.

Inferential and descriptive analyses were conducted on gliomas and meningiomas to examine tumor group, histological type, quality, clinical and surgical variables, and outcomes. Because the other tumors were extremely heterogeneous, they were excluded from the inferential analysis. Potential associations among outcomes, clinical/surgical variables, and differences in ECOG scores at admission and discharge were investigated.

We performed an additional analysis during the COVID-19 pandemic (between March 2020 and August 2021) to compare the patient numbers before and after the pandemic, ensuring that the pandemic did not influence the study’s results.

Parametric data, such as means and standard deviations, and non-parametric data, including medians and quartiles, were analyzed. The R computational language was used for all analyses with a 5% significance level. Pearson’s χ^2^, ordinal χ^2^, or Fisher’s exact tests were used to determine the associations between qualitative variables. Mann-Whitney, Kruskal-Wallis and Student’s *t*-tests were used to compare qualitative and quantitative variables. Dunn’s method was used to adjust p-values for multiple comparisons of significant results, and the marginal homogeneity test was performed to compare ECOG results at admission and discharge.

The Shapiro-Wilk test was used to verify the normality of the quantitative variables, whereas the correlation between continuous variables was assessed using Spearman’s correlation. A *t*-test was used to compare patient counts before and during the pandemic. In addition, Cox-Stuart and Fisher’s tests were used to check the significance of trends and seasonality.

Augmented Dickey-Fuller (ADF) tests were used to check the stationarity of the series using a graphical analysis of autocorrelation. The time-series breakpoints were verified using F-statistics. A p<0.025 in multiple comparisons suggests class differences. However, this analysis was not required for two-class variables. Variables such as “ASA: preoperative,” “ECOG: at admission,” and “ECOG at discharge” had fewer classes, which was due to limited patient numbers.

The study was approved by the Research Ethics Committee of *Hospital Israelita Albert Einstein* (CAAE: 30046420.9.0000.0071; # 4.122.528) and was conducted following the principles of the Declaration of Helsinki. Data were collected and analyzed using REDCap, and information confidentiality was ensured.

## RESULTS

In the present study, 356 patients with CNS tumors were selected, of whom 79% were scheduled for admission, 18.5% were admitted to the emergency department, and 2.5% were transferred to another center.

The study population comprised 52.7% females, with a median age of 53 years and an age range of ± 15 years. History of SAH, DLP, and DM were 34.9%, 21.2%, and 16.5%, respectively. Histopathological examination revealed gliomas in 172 cases (48.3%), whereas meningiomas and other tumors were found in 107 (30.0%) and 77 (21.6%) cases.

The quality measures proposed by the AAN/SNO, a multidisciplinary care plan, were implemented in 35% of patients with glioma (n=61). All patients with glioma underwent molecular testing (n = 172), and 130 received chemotherapy after providing full consent. Overall, 87% of patients (n=150) underwent postoperative cranial MRI within 72h, and 0.84% (n=3) experienced VTE events after primary brain tumor resection.

Prophylaxis for VTE was administered to 94.7% of patients with glioma, 94.3% of those with meningioma, and 97.4% of those with other tumors. Antiseizure medications were more prevalently used by patients with glioma (65.12%), whereas patients with meningioma commonly used corticosteroids (81.31%).

Regarding topography, deep intra-axial locations were the most prevalent in gliomas (51.1%), whereas extra-axial skull base anterior fossa locations were more common in meningiomas (30.8%). In other tumors, the sellar/suprasellar region was the most frequently observed site (54.5%). Notably, the glioma group commonly showed a tumor size >3cm (76.7%).

Among the groups, the preoperative ASA scores were II in 65.12% of gliomas, 60.75% of meningiomas, and 50.65% of other tumors. The ECOG performance scale scores were zero in 52.9%, 60.7%, and 63.6% of the patients in the respective groups. Notably, <40% of patients in all groups had neurological *deficits*. In the meningioma group, surgical extension was classified as radical in 61.6% of cases, whereas radical resection was achieved in only 18.6% of cases in the glioma group. Notably, 5.19% of patients with other tumors and 25.58% of patients with gliomas experienced epileptic seizures.

The average lengths of stay for patients with glioma, meningioma, and other tumors were 9, 8, and 7 days, respectively. A considerable proportion of patients (81.4%, 85%, and 68% for glioma, meningioma, and other tumors, respectively) had no postoperative complications. Notably, ECOG zero at medical discharge was more common across all tumor groups (58.7%, 60.7%, and 72.7% for glioma, meningioma, and other tumors, respectively). After 30 days, 81%, 87.85%, and 93.51% of patients with glioma, meningioma, and other tumors, respectively, were alive, with readmission occurring in six (3.49%), 12 (11.12%), and three (3.9%) patients, respectively.

**Figure 1:**
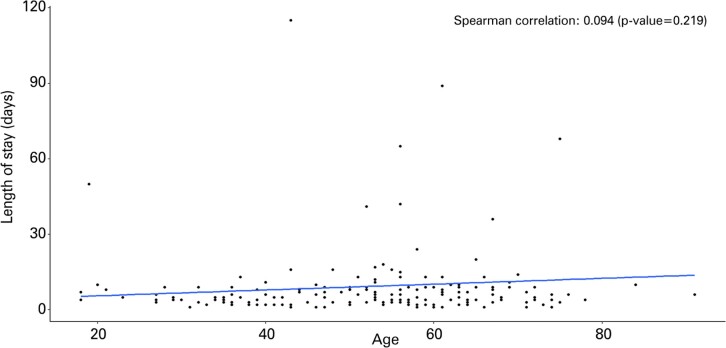
Spearman correlation between period of hospitalization and age of patients with glioma

**Figure 2:**
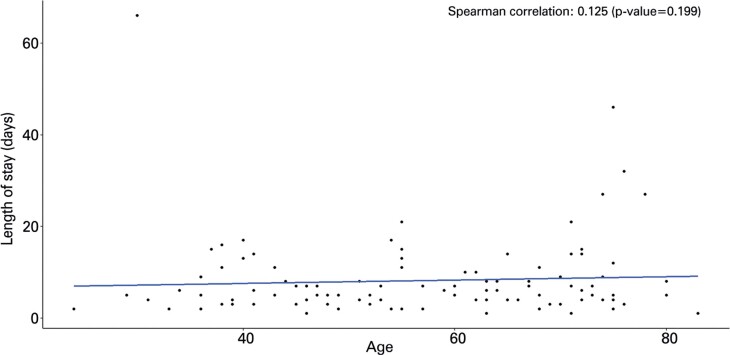
Spearman correlation between period of hospitalization and age of patients with meningioma

Our analysis revealed that age and length of hospital stay (in days) were not correlated in patients diagnosed with either glioma (Figure 1) or meningioma (Figure 2).

Based on the outcome variables, the length of stay was correlated with preoperative ASA, ECOG performance status at admission and discharge, and the presence of neurological deficits in gliomas. Additionally, the length of stay was associated with the ECOG performance status at discharge, the presence of neurological deficits, the size of the tumor, and the presence of epileptic seizures in meningiomas (Table 2).

**Table 2:** Length of stay (days) *versus* qualitative variables-glioma and meningioma

Variable	Class (number of patients)	Mean (SD)	p value	Class (number of patients)	Mean (SD)	p value
	**Glioma**			**Meningioma**		
ASA: preoperative	1 (34)	8.71 (14.78)	0.006	1 (25)	8.96 (12.65)	0.149
	2 (112)	6.54 (6.99)		2 (65)	6.82 (5.49)	
	3-4 (26)	22.35 (31.92)		3-4 (17)	12.12 (11.67)	
ECOG at admission	0 (91)	6.01 (6.98)	<0.001	0 (65)	7.91 (9.77)	0.312
	1 (62)	10.98 (19.07)		1 (36)	8.53 (7.87)	
	2-4 (19)	20.05 (27.35)		2-4 (6)	8.67 (3.27)	
ECOG at discharge	0 (101)	5.45 (3.87)	<0.001	0 (65)	6.05 (6.09)	0.001
	1 (31)	10.03 (19.88)		1 (16)	12.81 (14.98)	
	2 (25)	10.56 (14.22)		2-5 (26)	10.58 (8.53)	
	3-5 (15)	32.27 (33.51)				
Presence of neurological deficit*	No (103)	5.75 (5.26)	<0.001	No (70)	7.06 (8.99)	0.001
	Yes (69)	14.74 (23.4)		Yes (37)	10.24 (8.37)	
Tumor size*	<3cm (37)	8.16 (10.17)	0.598	<3cm (28)	5.29 (3.33)	0.015
	≥3cm (132)	9.85 (17.35)		≥3cm (70)	9.8 (10.38)	
Resection extension	Radical (32)	8.53 (7.19)	0.190	Radical (66)	8.06 (7.18)	0.572
	Partial (83)	9.7 (14.72)		Partial (23)	11.26 (13.9)	
	Biopsy (47)	10.7 (22.57)				
Presence of epileptic seizure*	No (128)	8.84 (14.76)	0.705	No (99)	7.12 (5.77)	0.041
	Yes (44)	10.86 (19.03)		Yes (8)	21 (22.87)	
Comorbidity*	No (114)	8.5 (14.39)	0.168	No (59)	7.8 (9.44)	0.263
	Yes (58)	11.03 (18.62)		Yes (48)	8.6 (8.19)	
Topography (Localization)	Intra-axial eloquent area or trunk (62)	10.55 (16.36)	0.463	Extra-axial cranial base anterior fossa (33)	9 (9.09)	0.536
	Deep Intra-axial (88)	9.27 (17.41)		Extra-axial cranial base posterior fossa (15)	6.47 (4.07)	
	Intra-axial polar (22)	6.32 (4.44)		Extra-axial cranial base medium fossa (16)	10.69 (15.75)	
				Extra-axial convexity without involvement of sinus venosus (17)	5.53 (4.64)	
				Extra-axial convexity with involvement of sinus venous (26)	8.23 (6.68)	

*Mann- Whitney U test or Kruskal-Wallis test (5% significance level). p-valor do Shapiro-Wilks >0.05.

SD: standard deviation; ECOG: Eastern Cooperative Oncology Group; ASA: American Society of Anesthesiologists.

The assessment of clinical and surgical variables and the “postoperative complication” outcomes in patients diagnosed with glioma showed that the ECOG at admission and discharge, the presence of neurological deficits, and epileptic seizures are associated with postoperative complications. Additionally, for patients with meningioma, ECOG performance status at discharge and epileptic seizures were associated with postoperative complications (Table 3).

**Table 3:** Postoperative complications *versus* qualitative variables - Glioma and Meningioma

Variable	Class (number of patients)	Yes (%)	p value	Class (number of patients)	Yes (%)	p value
	**Glioma**			**Meningioma**		
ASA: preoperative*	1 (34)	7 (21.88)	0.246	1 (25)	5 (31.25)	0.726
	2 (112)	16 (50)		2 (65)	8 (50)	
	3-4 (26)	9 (28.12)		3-4 (17)	3 (18.75)	
ECOG at admission*	0 (91)	12 (37.5)	0.033	0 (65)	11 (68.75)	0.936
	1 (62)	14 (43.75)		1 (36)	3 (18.75)	
	2-4 (19)	6 (18.75)		2-4 (6)	2 (12.5)	
ECOG at discharge	0 (101)	8 (25)	<0.001	0 (65)	5 (31.25)	0.028
	1 (31)	9 (28.12)		1 (16)	5 (31.25)	
	2 (25)	6 (18.75)		2-5 (26)	6 (37.5)	
	3-5 (15)	9 (28.12)				
Presence of neurological deficit*	No (103)	12 (37.5)	0.005	No (70)	8 (50)	0.235
	Yes (69)	20 (62.5)		Sim (37)	8 (50)	
Tumor size*	<3cm (37)	6 (18.75)	0.633	<3cm (28)	2 (14.29)	0.201
	≥3cm (132)	26 (81.25)		≥3cm (70)	12 (85.71)	
	Radical (32)	6 (18.75)	0.540	Radical (66)	8 (57.14)	0.285
Resection extension**	Partial (83)	19 (59.38)		Partial (23)	6 (42.86)	
	Biopsy (47)	7 (21.88)		0	0	
	No (128)	19 (59.38)	0.042	No (99)	12 (75)	0.017
Presence of epileptic seizure	Yes (44)	13 (40.62)		Yes (8)	4 (25)	
	No (114)	21 (65.62)	0.999	No (59)	11 (68.75)	0.281
Comorbidity	Yes (58)	11 (34.38)		Yes (48)	5 (31.25)	
Topography (Localization)	Intra-axial eloquent area or trunk (62)	12 (37.5)	0.862	Extra-axial cranial base anterior fossa (33)	6 (37.5)	0.858
	Deep Intra-axial (88)	15 (46.88)		Extra-axial cranial base posterior fossa (15)	1 (6.25)	
	Intra-axial polar (22)	5 (15.62)		Extra-axial cranial base medium fossa (16)	2 (12.5)	
				Extra-axial convexity without venous sinus involvement (17)	2 (12.5)	
				Extra-axial convexity with sinus venous involvement (26)	5 (31.25)	

Pearson’s χ^2^ test. * Ordinal χ^2^ test. ** Fisher’s exact test (5% significance level). p-valor do Shapiro-Wilks >0.05.

ECOG: Eastern Cooperative Oncology Group; ASA: American Society of Anesthesiologists.

The analysis of clinical outcomes, as measured by an ECOG score >1 at medical discharge, revealed a significant association with ECOG score at admission for both the glioma (p=0.001) and meningioma (p=0.0017) groups.

Regarding readmission within 30 days, none of the clinical and surgical variables analyzed in patients with gliomas were associated. However, for meningiomas, readmission was associated with ASA II (58.33%) and ASA III-IV (41.67%) (p=0.013).

The marginal homogeneity test was used to assess whether the paired proportions of patients with glioma and meningioma were dissimilar, indicating a potential improvement or deterioration in the patient’s condition. Rejection of the hypothesis of equal ECOG scores at admission and discharge for gliomas (p=0.036) and meningiomas (p=0.016) suggests changes in the patient’s condition.

Before the COVID-19 pandemic, 293 (82.3%) patients were admitted; however, only 63 (17.7%) were admitted during the pandemic. Our inferential analysis of the group variables (pre-pandemic *versus* pandemic) and the number of monthly patients showed no association between the groups (p=0.651).

## DISCUSSION

In this study, we highlighted the importance of quality assessment in neuro-oncology following the AAN/SNO guidelines and suggested new indicators for evaluating clinical practice, monitoring professional performance, and enhancing care quality.

### Statement of principal findings

Multidisciplinary discussions, including specialists in neuro-oncology, neurosurgery, oncology, neuroradiology, and neuropathology, are crucial for creating care plans and establishing brain tumor centers. Reportedly, these discussions result in better quality and coordination of care for various cancer types, making them key quality indicators in cancer care both nationally and internationally.^16^ In the HIAE, these discussions have been held since 2008 and have covered almost 35% of the eligible cases. However, considering that participation is voluntary and patients should be referred by autonomous medical staff for discussion is important.

In the 2016 WHO classification of CNS tumors, molecular characteristics, such as isocitrate dehydrogenase (IDH) wild-type and IDH-mutated glioblastomas, were significantly incorporated.^19^ For tumors lacking molecular diagnostic testing, a “not otherwise specified” (NOS) designation was permitted. In the 2021 update, CNS 5, new nomenclature and classification approaches were included, which emphasized integrating molecular diagnosis with morphology and introduced new tumor types and subtypes. Arabic numerals for tumor grading 1-4 were also implemented.^20^ Molecular diagnoses, which are crucial for better diagnosis and prognosis of gliomas and for several primary nervous system tumors, were fully incorporated in the 2021 classification.^20^

The AAN/SNO recommends performing molecular testing for grade 1-4 gliomas. This was based on the 2016 WHO classification as CNS 5, which was only published in 2021.^20^ In the present study, 100% (n=172) of the glioma patients were anatomopathologically diagnosed following the 2016 classification, considering the study period was between 2015 and 2021.

The guidelines of the American Society of Oncology and Oncology Nursing recommend patient education before chemotherapy is prescribed.^13^ Meanwhile, SNO/AAN recommends obtaining informed consent from patients or their representatives for ethical and high-quality cancer care and potential legal risk mitigation. Best practices involve written consent signed by the patients or their representatives.^16^ In this study, all patients with chemotherapy signed a consent form.

Neuroimaging is crucial in the diagnosis, surgery planning, and treatment of CNS tumors. This is particularly significant for gliomas, in which the extent of resection affects survival. Postoperative imaging is essential for oncologists to devise effective treatment plans and conduct clinical trials requiring residual disease measurements.^22^ Guidelines recommend performing postoperative MRI within 72h after resection to counter vascular and inflammatory changes that might affect imaging specificity. We observed a high level of adherence to this measure in our study, with 87% of patients receiving the recommended imaging.

Common complications in cancer cases, particularly those with CNS tumors, include thromboembolic events, such as pulmonary embolism and deep vein thrombosis. Compression stockings are often used as a low-risk option to prevent VTE during the perioperative period.^16^ Two prospective clinical trials showed that adding low-molecular-weight heparin to pneumatic devices increases their safety and efficacy.^16^ However, most patients do not receive chemoprophylaxis after brain tumor surgery. At the HIAE, there was an institutional VTE management protocol that probably resulted in a low incidence of VTE, which affected only three patients (0.84%).

The proposal included outcomes to evaluate potential quality indicators for CNS tumors, such as length of hospital stay, postoperative complications, ECOG performance status at discharge, and 30-day readmission. Inferential analysis revealed that ASA and ECOG findings at admission and discharge, as well as neurological deficits, were associated with the length of hospital stay in patients with gliomas. However, no variables were associated with the 30-day readmission rates. The significant variables observed in the postoperative complication analysis were ECOG status at admission and discharge, neurological *deficit*, and epileptic seizures.

The length of stay for patients with meningioma was influenced by the ECOG performance status at discharge, neurological deficits, tumor size, and epileptic seizures, with ASA linked to the 30-day readmission. Postoperative complications were significantly associated with ECOG status at discharge and epileptic seizures.

The pandemic did not affect patient admissions when compared to the pre-pandemic data, as seen in the glioblastoma study by Neff et al.^23^ Despite the distinct pre-pandemic (5 years) and post-pandemic (1.5 years) periods, the homogeneity hypothesis holds for neurological deficits and tumor size, suggesting that patient’s diagnosis were not delayed despite pandemic challenges.

### Strengths and limitations

In this study, challenges were encountered in measuring treatment-related data, such as chemotherapy or radiotherapy, owing to limited access to health insurance and variations in decision-making among the open clinical staff during follow-up and relapse. These factors, combined with the complexity of treating intracranial metastases that require targeted therapies and immunotherapy, were excluded from this study. Despite these limitations, this study represents a pioneering effort to enhance the management of patients with neuro-oncological disorders within institutions and establish the quality of care provided.

### Interpretation within the context of the wider literature

Riblet et al.^15^implemented quality measures for patients with glioma at the Dartmouth-Hitchcock Medical Center. These measures, including notification of patient admission, neuro-oncology evaluation, social worker assessment, and post-discharge follow-up, have improved real-time patient navigation and care quality. Despite including other tumor types in this study, further improvements are needed in real-time glioma management, particularly in metric adherence monitoring and organized post-discharge follow-up.

Silvestre et al.^18^ studied the implementation of quality measures in patients with brain metastasis (BM) at the University of Virginia Health System. They emphasized that patients with BM are distinct from those with primary tumors because of their unique metastatic characteristics. Some measures, such as the survival rate of >90 days and the proportion of patients alive at 90 days postoperatively, could be relevant to our institution. These measures offer insights into the surgical quality, patient severity, and long-term prognosis. Palliative care was also identified as a significant factor.^18^ We also examined data on 30-day hospital readmission, categorizing it as related to either admission diagnosis or elective readmission. However, a 90-day evaluation would require an improved service organization for long-term case monitoring, which is currently not feasible because of patient and structural constraints.

Vanhauwaert et al.^24^ used the Delphi method to evaluate CNS tumor patient care and identified seven key areas: diagnosis, surgery, pathology, radio/chemotherapy, recurrence, supportive care, and survival.^24,25^ This study was still in the final data collection stages when these areas were monitored. However, there were limitations to data collection for radiotherapy/chemotherapy due to patient insurance coverage restrictions. Rehabilitation was also not measured in this study, thereby missing insights into tumor-related disabilities and their impact on patient outcomes.

Our institution has adopted the 2021 NICE guidelines for managing brain tumors and metastases; hence, our study proposals aligned with these guidelines. Recommended protocols have advanced our diagnostic and resection techniques, and molecular markers have been used for glioma prognosis or treatment. Despite the progress in the early diagnosis and treatment of gliomas, improvement in long-term patient monitoring and referral visibility is needed, and providing optimal care for patients with neuro-oncology is necessary.

### Implications for policy, practice, and research

The use of prospectively reported performance measures allows a multidisciplinary team to visualize evidence of the work process and establish new indicators. This approach enables professionals to feel involved in the process and be accountable for its results, leading to increased protagonism and positive outcomes for patient care and their families. Moreover, offering surgical packages based on the histological type of the tumor, such as standardizing clinical and surgical practices through bundles, would improve the predictability of intrahospital costs, including expenses for hospitals, patients, and healthcare providers. However, significant variability in surgical costs was observed during the case analysis period, such as using different materials intraoperatively for the same tumor type owing to individualized care provided based on the specificities, severity, and location of the tumor and the autonomy of the open clinical staff.

## CONCLUSION

The medical team showed a praiseworthy commitment to the quality standards outlined by the American Academy of Neurology and Society of Neuro-Oncology. Nevertheless, there are areas for enhancement, particularly in the multidisciplinary care plan and execution of postoperative imaging examinations.

Moreover, the duration of hospital stay, postoperative complication incidence, Eastern Cooperative Oncology Group evaluation at discharge, and status within 30 days can serve as quality measures. These should be adjusted for the American Society of Anesthesiologists and Eastern Cooperative Oncology Group scales and should account for the occurrence of epileptic seizures and neurological deficits, specifically for meningiomas and tumor size. These findings underscore the significance of incorporating a quality improvement program to achieve the strategic goal of delivering best-care medical practices.
